# Supplementary material for: Northern shrimp Pandalus borealis population collapse linked to climate-driven shifts in predator distribution
Source: PLoS One. 2021 Jul 21;16(7):e0253914. doi: 10.1371/journal.pone.0253914 (PMC8294506; doi:10.1371/journal.pone.0253914)
Supplement: S1 Appendix — Species scientific names are given in S2 Appendix. (DOCX) [file pone.0253914.s001.docx]

S1 Appendix. Summary of results of analysis of changes in predation. Species scientific names are given in SI2_Appendix.

| Predation analysis | Species considered | Species selected |  |  |  |  |
| --- | --- | --- | --- | --- | --- | --- |
|  |  | Spring offshore | Spring inshore | Summer offshore | Fall offshore | Detail |
| Increased biomass of other species (potential predators) | 59-99 species | (none) | Atlantic halibut  Longfin squid  Windowpane | Longfin squid | Atlantic mackerel  Longfin squid  Windowpane | Table 2, Fig 7, SI2_Appendix |
| Query of food habits database for Pandalid consumption by all species sampled (to find previously un-identified predators) | Acadian redfish  American plaice  American shad  Atlantic cod  Atlantic halibut  Atlantic herring  Atlantic mackerel  Atlantic wolffish  Barndoor skate  Black sea bass  Blueback herring  Butterfish  Cunner  Cusk  Fourbeard rockling  Fourspot flounder  Goosefish  Gulf stream flounder  Haddock  Little skate  Longhorn sculpin  Northern searobin  Ocean pout  Pollock  Red hake  Scup  Sea raven  Silver hake  Smooth skate  Spiny dogfish  Spotted hake  Thorny skate  White hake  Windowpane  Winter flounder  Winter skate  Witch flounder  Yellowtail flounder | No new predators | No new predators | No new predators | No new predators |  |
| Change in consumption of Pandalids by known fish predators (PFO and PW) | Acadian redfish  American plaice  Atlantic cod  Atlantic halibut  Atlantic herring  Fourspot flounder  Haddock  Little skate  Longhorn sculpin  Monkfish  Pollock  Red hake  Sea raven  Silver hake  Smooth skate  Spiny dogfish  Thorny skate  White hake  Windowpane  Winter skate  Atlantic mackerel | Atlantic cod (PW) | (none) | (none) | (none) | Fig 8 |
| Local overlap with known predators | Acadian redfish  Atlantic cod  Atlantic mackerel  Longfin squid  Silver hake  Spiny dogfish  White hake  Windowpane | Longfin squid | Longfin squid | Longfin squid | Longfin squid | Fig 9 |
| Global overlap with known predators | Acadian redfish  Atlantic cod  Atlantic mackerel  Longfin squid  Silver hake  Spiny dogfish  White hake  Windowpane | (none) | Acadian redfish | Atlantic cod Longfin squid Silver hake  Spiny dogfish White hake | Atlantic mackerel  Longfin squid | Fig 10 |
