# Supplementary material for: Northern shrimp Pandalus borealis population collapse linked to climate-driven shifts in predator distribution
Source: PLoS One. 2021 Jul 21;16(7):e0253914. doi: 10.1371/journal.pone.0253914 (PMC8294506; doi:10.1371/journal.pone.0253914)
Supplement: Attachment — Submitted filename: response to reviewers.docx [file pone.0253914.s015.docx]

Richards and Hunter, Northern shrimp *Pandalus borealis* population collapse linked to climate-driven shifts in predator distribution

We very much appreciate the detailed and careful review. Our responses are highlighted in gray below each comment. A checkmark (🗸) indicates a minor comment taken care of with no textual response here.Comments from the editor:

We note that you have indicated that data from this study are available upon request. PLOS only allows data to be available upon request if there are legal or ethical restrictions on sharing data publicly.

We have added raw data to supporting information where appropriate, all other data are included as typically presented in this field, i.e. as calculated values (indices) rather than raw data.

In particular in your revision try to address in your discussion some reasons/thoughts on why the population has not recovered in recent years; and include also some information on recruitment processes in relation to the stock SSB.

We have expanded our discussion of this topic (paragraph starting line 587).

Our revised conflict of interest statement is as follows:

“The authors have read the journal’s policy and have the following conflicts: membership in a government advisory board (RAR and MH). This does not alter our adherence to PLOS ONE policies on sharing data and materials.”

Regarding copyright issues for Figures 1, 6 and 13:

Figures 1, 6 and 13 were created for this manuscript, and therefore do not require permission.

Comments from the reviewer:

Reviewer #1: INTRODUCTION
🗸 In the introduction, it would be useful with some more details on the marine heatwave occurring in 2012. What temperatures occurred, where (bottom, surface, all layers?), and when (spring, summer?).

🗸 The first time “Gulf of Maine” is mentioned in the text it should be followed by the abbreviation GOM in brackets, thereafter the abbreviation should be used (exceptions figure and table legends, and abstract). In the introduction, GOM and Gulf of Maine are used interchangeably.

🗸 Line 61: remove “)” after GOM

Line 76-77: Re the sentence “age assignments based on length, life history stage and reproductive status”, how can age be determined based on life history stage and reproductive status, as for instance males and berried females may consist of at least two year-classes: I would suggest to just write “age assignments based on length”.

We have removed the reference to reproductive status to generalize this statement, and added a citation that explains how the difference between first year and second year females can be determined when they are not berried. The fall survey is the only one in which females are berried and life history stage (male, female I, female II) cannot be used to help ‘age’ the shrimp.

MATERIALS AND METHODS
🗸 Line 109-110: Text reads “The inshore survey data included depths from ~40 m to 120 m …” while Table 1 says ~37 m to 120 m. The same interval should be used in text and Table 1.
Line 110: “… spatial overlap analysis, 5-120 m; …” it is not clear what data are used in the spatial overlap analysis, I cannot understand that there exist trawl data from depths of 5 m!

We have clarified the inshore depths and edited Table 1. A relatively small vessel (54 ft length overall) is used for the inshore surveys; it can tow in fairly shallow water.

🗸 Table 1, last line and first column in Table should be: “ASMFC and NEFSC summer” as it is written earlier in text: “… summer offshore surveys jointly by the Atlantic States Marine Fisheries Commission (ASMFC) and NEFSC.”

🗸 The summer offshore survey is a dedicated shrimp survey. Please insert a sentence specifying what the target species of the other surveys are (or perhaps ecosystem surveys?) It says in caption of Fig. 2 that they are “resource” surveys, this should be stated in M&M.

🗸 Lines 129-130: In this sentence “NEFSC offshore survey methods were modernized in 2009 with a new survey vessel, a modified trawl net design and shorter tow duration (Table 1; [37])” I would include also “different trawling speed”.

🗸 Line 145: should the year interval 1991-2004 include also 2005? As the text states that shrimp was not identified to species for the years 2006-2012.

🗸 Line 148: Insert a sentence stating how sampling of shrimp for length and stage determination was carried out on the spring inshore and summer offshore surveys. How many shrimps were sampled and measured per trawl station, and were shrimp sampled at all trawl stations?

🗸 Lines 152-153: were the “reference annual cycle” daily means of temperature recorded in the period 1978-1987? I suggest to include a brief description of the “reference annual cycle”

🗸 How was sea surface temperature measured? This is not mentioned in the M&M, but sea surface temperature anomalies are shown in Fig. 4.

🗸 I assume bottom temperatures from the inshore and offshore summer surveys also are from CTD measurements, this should be explicitly mentioned.

Line 163: Transition to spring and fall: what time period (years) are incorporated in the “average annual temperature”?

The reference period here was 1982-2017. We have added this to the text.

🗸 Lines 218-219: what is the difference between “bivalves” and “bivalve mollusks”?

🗸 Line 231: Bis should be in italics

🗸 Lines 242- : Re stomach data sampling, it would be useful with a sentence stating if stomach sampling was carried out for all fish species in the catch.

🗸 Line 267: “I” is from Eq. 3, not 2.

🗸 Line 273-274: the extra shallow strata in the inshore survey is here said to cover depths between approx. 9 and 37 m, while in line 110, the depth interval is given as 5-37 m. The same interval should be used.

This was due to a failure to convert from fathoms (5 fa) to meters (9 m) for the shallowest depths. We have fixed this throughout.

A lot of fish species have been investigated in this work, and it is hard to keep track of which are important where (stomach sampling of new species, stomach sampling of already identified shrimp predators, biomass peaks in 2012, spatial overlap studies). I had to read the M&M and Results section several times to get an overview. Would it be an idea to include a Table in the M&M that gives an overview including number of investigated species per topic, and number of resulting “interesting” species presented in results/Figures, and also a list of the names of these “interesting” species, per topic.

Yes, good idea. We’ve added a summary table as Supporting Information for the analysis of changes in predation (SI1_Appendix).

RESULTS
Fig. 2 should have error bars, but I do see the dilemma as these might make the figure less readable as the bars (means) for the years with lowest densities will be even smaller, in order to include error bars for the highest values.

This is a good point. We have added average coefficients of variation to the figure legend to give an indication of the variability in each of the survey indices. However, CVs were not calculated for the spring offshore survey because of the complicated manner in which indices needed to be estimated (as described in methods).

🗸 Fig. 3: text in figure is small and very hard to read.

🗸 Fig. 4 legend: should include the abbreviations BTA and SSTA. I suggest writing: (A) Bottom and sea surface temperature anomalies (respectively BTA and SSTA) for NEFSC spring and fall surveys

🗸 Somewhere there should be included some information on long-finned squid. How large do they grow, i.e. is it likely that they will prey on both male shrimp and the large females? Are they benthic or pelagic or both?

🗸 Fig. 7, legend should explain the hatched line = 0.85; that species were selected for further consideration if they had a standardized biomass index above 0.85 in 2012.

Fig. 8, the circles and squares are difficult to tell apart in the figure. I also wonder about the statement: “The only species that was an outlier in terms of percent of stomach content by weight (PW) in 2012 was Atlantic cod in spring”, as I don’t see an outlier in the cod-graph in 2012. The only high frequency (PW) in 2012 seems to be for smooth skate.

We have modified the figure to make the symbols easier to distinguish.

The statement about cod PW in spring 2012 is correct. Because the y-axis scales are standardized across sub-plots, it’s hard to see that the value for PW for cod in spring 2012 looks like (and is) an outlier. The variability in the smooth skate PW time series is generally higher, so the 2012 value does not stand out as an outlier in the series (visually or statistically). The 2007 value for smooth skate is an outlier by our definition (mean +/- 2 SD), but not 2012. We’ve added text so readers can calculate the outlier value for Atlantic cod for themselves if so desired without referring to the supplemental information.

🗸 Lines 383-385: This sentence talks about 10 fish predators, while Fig 8 shows data from 11 species: “Pandalid shrimp did not occur more frequently in stomach contents (PFO) of the 10 fish predators with sufficient sample sizes during NEFSC spring or fall offshore surveys in 2012 (PFO, Fig 8).”

Line 401: spatial overlap. I wonder why smooth skate was not considered for spatial overlap as this species had rather high percentage of shrimp in its diet (Fig8) as well as higher biomass index in 2012 compared to for instance cod (Fig7), which was included in the spatial overlap analysis.

Our reply above to the comment on Figure 8 (above) is relevant here. Also, the smooth skate PW was relatively high only offshore in the spring of 2012, and PFO was not high. Smooth skate biomass was not high during 2012. The biomass index values are not directly comparable between species because they are relative biomass indices, not absolute biomass estimates (e.g. 1 kg/tow of cod does not represent the same absolute biomass as 1 kg/tow of smooth skate because they undoubtedly have different catchabilities in the survey). We have added the term ‘relative’ to the figure captions as reminders.

🗸 Fig 11. I wonder how F can be given on an axis from 0 to 20. F is normally a value between 0 and 1.

These are not absolute estimates of F, but are ‘relative F’ as defined in equation 6. F_rel_ (and absolute F) can exceed a value of 1.

DISCUSSION
🗸 Line 463-465: “estimates were also very high during 1995-2001, exceeding the 2011-2012 values in two of the three models evaluated, with no concomitant collapse of the population.” Here, I think it should be noted that although the population did not collapse, it did however, reach the same low level as in 2012 (Fig.11) (at least the SSB).

The point we are trying to make here is about relative F, which depends on both SSB and landings. SSB by itself is not entirely relevant here, but we appreciate this comment, and have added discussion relevant to it in response to a similar comment below (see paragraph starting on line 587 of ms).

Fig 12: this figure should be extended until present. If temperature data are not available, at least the landings data of shrimp and squid should be presented.

We have carefully considered this comment and in the end decided to not add another plot (plots) because the analysis and interpretation becomes a lot more complicated with the development of management controls, changes in fishing effort, confidentiality of some landings data, and the proliferation of not-entirely-continuous fishery-independent indices starting in the late 1960s. These could be added, but our sense is that a deeper look would require significantly more discussion that would add to an already rather lengthy paper, and would probably be best left for another paper.

GENERAL COMMENTS ON MS, ESPECIALLY DISCUSSION
This is very interesting work, and the issues explored are impressing. The introduction is well written and gives a good overview of the background for the research presented. A lot of species and topics were explored and it is hard to get an overview, thus in the methods section I suggest an overview table. The results are well presented and well discussed. Below are some points for consideration.

The authors talk about the population collapse happening in 2012, but might it be more correct to talk about a collapse happening over the two years 2012-2013, as the really low population size was reached in 2013?

This is a valid point and we have gone back and forth on it. The indices did drop again from fall of 2012 to spring of 2013, and remained at essentially the same low levels thereafter. In the end we chose to call it 2012 for simplicity, and because this was when the first major decline occurred. However we have added text throughout referring to early 2013 to bring out the point that the decline also included early 2013.

It would be interesting if the authors in the discussion would comment on the fact that the population has remained in a collapsed state also after 2012-2013. And discuss possible causes. Fig 11 shows that the population in 2001 (SSB) was at the same low level as in 2012. However, at that time the population quickly recovered. Why has the population not recovered in recent years? This should be touched upon in the discussion. Does the squid continue to exert a strong predation pressure on the shrimp? Is the bottom temperature still at record high levels? Is spring still occurring earlier than usual? Or is the shrimp population now at such low levels (below a not-specificed Blim-level) that recruitment is impaired?

We have added discussion to address this comment in the paragraph starting on line 587.

Information on the recruitment to the stock is missing, other then what can be read from Fig. 3. It would be useful with a plot of the time-series of recruitment (age 1 shrimp). As a short-lived species, the size of the P. borealis stock is highly dependent on the incoming recruitment. Thus, the size of the stock in 2012 was influenced also (in addition to increased predation) by the low recruitment in 2011 leading to low numbers of 2-year old males in 2012.

We have added a panel showing recruitment indices in Figure 2.

Many thanks to this reviewer for the careful and helpful job in combing through a lot of material in this paper.
